# Optimization of Enzyme-Assisted Extraction of Rosemary Essential Oil Using Response Surface Methodology and Its Antioxidant Activity by Activating Nrf2 Signaling Pathway

**DOI:** 10.3390/molecules29143382

**Published:** 2024-07-18

**Authors:** Yuanyuan Li, Lei Huang, Yongfang Xu, Biao Cheng, Mingqin Zhao

**Affiliations:** 1 Flavors and Fragrance Engineering & Technology Research Center of Henan Province, College of Tobacco Science, Henan Agricultural University, Zhengzhou 450046, China; 2 School of Pharmacy, Zhengzhou Shuqing Medical College, Zhengzhou 450064, China

**Keywords:** *Rosmarinus officinalis* L., essential oils, response surface methodology, antioxidant activity, Nrf2 signaling pathway

## Abstract

Rosemary essential oil (REO) is widely recognized as a food flavoring and traditional herb and possesses potential antioxidant activity. However, its low yield rate and unclarified antioxidant mechanism warrant further investigation. In this study, an enzyme pretreatment-assisted extraction method with Box–Behnken design (BBD) and response surface methodology (RSM) models was employed to optimize the main factors of REO, and its antioxidant molecular mechanism under oxidative stress was elucidated in hydrogen peroxide-induced human lung carcinoma (A549) cells. The optimized yield (4.10%) of REO was recorded with the following optimum conditions: enzyme amount 1.60%, enzyme digestion pH 5.0, enzyme digestion temperature 46.50 °C, and enzyme digestion time 1.7 h. Meanwhile, 1.8-cineole (53.48%) and β-pinene (20.23%) exhibited radical scavenging activity higher than that of BHA and BHT. At the cellular level, REO (12.5–50 µg/mL) increased the levels of cell viability, CAT, SOD, and GSH significantly while reducing the contents of ROS, MDA, and GSSG, when compared to H_2_O_2_ exposure. Mechanically, REO relieved oxidative stress via activating the Nrf2 signaling pathway and enhancing the protein expression of Nrf2, NQO-1, and HO-1, which was further verified by molecular docking between the main component 1.8-cineole and the Kelch domain of KEAP1. Therefore, REO could be considered as a potent natural antioxidant with a potential strategy in the food and pharmaceutical industries.

## 1. Introduction

Essential oils are complex liquids comprising a multitude of volatile and non-volatile compounds with an aromatic odor. They have been employed in a variety of applications in the pharmaceutical and natural therapies industries, as well as the food industry, since ancient times [1]. *Rosmarinus officinalis* L., commonly known as rosemary, is an evergreen aromatic plant belonging to the Lamiaceae family [2]. Rosemary has been widely recognized as an appreciated medicinal plant and evidenced to restore redox equilibrium due to its abundant content of essential oil. Meanwhile, REO has been employed as a flavoring and antioxidant in the food preservation and cosmetics industries [2,3]. It is increasingly acknowledged in the nutrition industry for food preservation and stability, as well as oxidative stress mitigation [4,5]. Due to the high demand for REO, there is a growing concern regarding the improvement of yield mainly extracted by steam distillation, microwave extraction, and supercritical fluid extraction [6]. Enzyme-assisted extraction, known as a novel and efficient method for extracting essential oils from aromatic plants over conventional methods including steam distillation or solvent extraction, offered higher yield, shorter extraction times, environmentally friendly processes, and potentially better preservation of bioactive compounds due to milder extraction conditions [7,8]. Nevertheless, the literature is limited to the optimization of enzyme-assisted extraction of REO, and the mechanisms accountable for its antioxidant properties are rarely identified.

Countless evidence supports that oxidative stress plays a critical mediator in the transduction of pulmonary disorders, which were the earliest pathophysiological mechanism described in association with air pollution exposure (cigarette smoke, air pollutants, exhaust fumes) [9]. The excessive reactive oxygen species (ROS), including H_2_O_2_, O_2_^•−^, •OH, and 1O2, derived from the burden of air pollution exposure, have been identified as a critical triggering event. This imbalance between the production of pro-oxidants and the capacity of antioxidants to neutralize them has been considered to be a key factor in the pathogenesis of pulmonary disorders [10,11]. Considering the pathological role of ROS, current studies are focused on the pursuit of effective antioxidant sources, which are defined as a defense system scavenging the surplus of free radicals. Nuclear factor erythroid 2-related factor 2 (Nrf2) performs a pivotal role in the transcriptional regulation of genes encoding antioxidant proteins, which are implicated in glutathione synthesis, xenobiotic detoxification, and the disposal of ROS [12]. In particular, essential oils derived from medicinal and aromatic plants have been employed as antioxidant alternatives due to their substitution, and their antioxidant has been suggested to activate the Nrf2 signaling pathway [13]. However, it is still unknown whether REO exerts its antioxidant effects through the activation of the Nrf2 signaling pathway. 

Therefore, the present study aimed to optimize enzyme-assisted extraction of REO using response surface methodology and to investigate its antioxidant activity by activating the Nrf2 signaling pathway. In this study, the effects of enzyme amount, enzyme digestion pH, enzyme digestion temperature, and time on the yield of REO via enzyme-assisted extraction were evaluated by employing response surface methodology. Subsequently, the composition of the extracted REO under the optimal process conditions was analyzed by GC-MS. Furthermore, the antioxidant activity of REO was evaluated utilizing radical scavenging assays, and the underlying antioxidant mechanisms were investigated in hydrogen peroxide-induced A549 cells. 

## 2. Materials and Methods

### 2.1. Experimental Materials

GC–MS analysis was conducted on an Agilent 7890 gas chromatography mass spectrometer (Agilent, Santa Clara, CA, USA) equipped with a flame ionization detector (FID, Agilent Corporation, Santa Clara, CA, USA). BHA (betahydroxy acid), BHT (butylhydroxytoluene), ascorbic acid (Vitamin C, VC), DPPH (1,1-Diphenyl-2-picrylhydrazyl radical 2,2-Diphenyl-1-2,4,6-trinitrophenyl hydrazyl), and ABTS (2, 2′-azino-bis-3-ethylbenzothiazoline-6-sulfonic acid) were provided by Sigma Chemical Co. (St. Louis, MO, USA). Reactive oxygen species assay kit, RIPA (Radio immune precipitation assay) lysis buffer, SOD (Superoxide dismutase), GSH (glutathione), MDA (Malonaldehyde), and CAT (Catalase) assay kit were bought from Beyotime Biotechnology (Beyotime, Shanghai, China). The antibodies of Nrf2 (Nuclear factor erythroid 2-related factor 2), NQO1 (NAD (P)H:Quinone Oxidoreductase 1), HO-1 (Heme Oxygenase-1), GAPDH (glyceraldehyde-3-phosphate dehydrogenase), and HRP (horseradish peroxidase)-conjugated secondary antibodies were obtained from CST (Cell Signaling Technology, Beverly, MA, USA). All of the reagents and solvents were purchased from Sinopharm Chemical Reagent Co., Ltd., (Shanghai, China) unless otherwise specified.

### 2.2. Extraction of Rosemary Essential Oil

Rosemary was collected from Yuzhou Country, Xuchang City, Henan Province, China. An average was obtained in September 2022. The voucher specimens (HAU 450066) were stored in the Flavors and Fragrance Engineering & Technology Research Center. Acidic cellulase (>10,000 U/g) enzyme was purchased from Sigma-Aldrich Inc. (St. Louis, MO, USA) and dissolved in citrate-phosphate buffer at pH 5.0. The enzyme-assisted extraction was conducted in two stages, including enzymatic pre-treatment and further extraction by hydrodistillation. Initially, rosemary (overground part) was pulverized into particles and pulverized into a homogeneous size by a disintegrator (50–60 mesh). Subsequently, 100 g homogeneous rosemary powder was combined with 500 mL of a 2.0% cellulase enzyme solution (citrate-phosphate buffer at pH 5.0) and further incubated prior to being subjected to hydrodistillation. After that, extraction was conducted in a Clevenger apparatus, followed by steam distillation for 4 h. After that, the distilled essential oil was stored at 4 °C after being dried with anhydrous sodium sulfate.

### 2.3. Experimental Design

#### 2.3.1. Single-Factor Experiments of Enzyme Amount, Enzyme Digestion pH, Enzyme Digestion Temperature, and Time 

In single-factor experiments, four variables including enzyme amount (0.5~2.5%), enzyme digestion pH (4.0~6.0), enzyme digestion temperature (35~45 °C), and enzyme digestion time (0.5~2.5 h) were investigated to assess the comparatively significant variables for the yield of REO.

#### 2.3.2. Box–Behnken Design

The Box–Behnken design model was used to optimize the enzyme-assisted extraction conditions of REO. Four independent variables are shown in Table 1, assigned −1 and +1 for low and high values (Table 1). 

RSM was designed with the independent variables of enzyme amount, enzyme digestion pH, enzyme digestion temperature, and time, with REO yield as the evaluation target. The predicted REO yield from twenty-nine experiments was calculated using Design Expert 13 software and is presented in Table 2. The yield of REO was evaluated by the following second-order polynomial equation. Analysis of variance was used to determine the significant differences in the yield of REO under different conditions.

### 2.4. GC-MS Analysis of Rosemary Essential Oil

The quantification analysis of REO was conducted on an Agilent 7890 gas chromatograph-mass spectrometry (GC-MS) equipped with an HP-5ms capillary column (30 m × 0.25 mm, 0.25 μm, Thermo Fisher Scientific Inc., Waltham, MA, USA). The REO sample was prepared with 1 mg/mL diluted with CH_2_Cl_2_. The initial column temperature was set at 60 °C for 2 min, raised to 180 °C at 10 °C/min, held for 1 min, then boosted to 260 °C at 20 °C/min, and maintained for 15 min. The injector temperature was kept at 250 °C and the detector (FID) temperature at 280 °C. The carrier gas (helium) was 1.0 mL/min. An injection volume of 1 µL was injected at a split ratio of 1:100. Mass spectrometry was performed in full scan mode ranging from 50 to 550 *m*/*z*. The ionization voltage was set at 70 eV. The components of REO were identified by comparing the mass spectra with those in the National Institute of Standards and Technology (NIST17) mass spectra libraries and calculating the experimental GC retention indices (RI) using a mixture of homologous series of normal alkanes C7~C40 in hexane. The proportions of each constituent were determined by considering the individual and total peak areas in triplicate (*n* = 3) and expressed as mean ± standard deviation.

### 2.5. Antioxidant Activity of Rosemary Essential Oil

#### 2.5.1. DPPH Radical Scavenging Activity of Rosemary Essential Oil

The free radical scavenging activity of REO was assessed using the methodology previously described by Li et al. [12]. Briefly, a series of concentrations of REO (12.5–1000 μg/mL) or positive standard BHA, BHT, and ascorbic acid (12.5–200 μg/mL) were combined with *DPPH* (0.2 mM) in a 96-well plate with a total volume of 150 μL. After incubation at room temperature for 30 min in the dark, the absorbance was determined at 520 nm. Methanol was used instead of the sample in the blank control. The formula used to determine radical scavenging activity was as follows:

$$DPPH\;radical\;scavenging\;activity\;\left(\%\right)=\left[1-\left(A_{1}-A_{2}\right)/A_{0}\right]\times 100\%$$

*A*_1_ was recorded as the absorbance of REO. *A*_2_ was recorded as the absorbance of methanol instead of *DPPH*. *A*_0_ was recorded as the absorbance of methanol. Experiments were conducted in triplicate.

#### 2.5.2. *ABTS*^+^ Radical Scavenging Activity of Rosemary Essential Oil

The *ABTS* assay was conducted according to Re et al. [14] with minor modifications. Briefly, the solution of *ABTS*^+^ was combined with 5 mL of 7 mmol/L *ABTS*^+^ and 88 µL of 140 mmol/L potassium persulfate. The reaction was conducted for 12 h at room temperature in the dark. 

Approximately, 2 mL of REO (62.5–1000 μg/mL) was mixed with *ABTS*^+^ solution (2 mL) and allowed to react for 6 min in the dark. Absorbance was measured at 734 nm, with BHA, BHT, and ascorbic acid as positive group. The formula used to determine the radical scavenging activity was as follows:

$$ABTS^{+}radical\;scavenging\;activity\left(\%\right)=\left[\left(A_{0}-A_{s}\right)/A_{0}\right]\times 100\%$$

The absorbance of REO was recorded as *A_s_*. *A*_0_ was recorded as a blank control by using ethanol instead. Experiments were conducted in triplicate.

#### 2.5.3. Hydroxyl Radical Scavenging Activity of Rosemary Essential Oil

The hydroxyl radical scavenging activity was assayed according to the method described in previous reports by Zhu et al. [15]. A mixture of 5.0 mM 1,10-phenanthroline (600 μL), 5.0 mM FeSO_4_ (600 μL), and 15 mM EDTA (600 μL) was combined with 0.2 M sodium phosphate buffer (400 μL, pH7.4). REO (600 μL) at 12.5–1000 μg/mL and 0.01% H_2_O_2_ (800 μL) were then combined and incubated at 37 °C for 60 min, after which the absorbance was read at 540 nm. BHA, BHT, and VC were used as positive control. The formula used to calculate hydroxyl radical scavenging activity was as follows:

$$Hydroxyl\;radical\;scavenging\;activity\;\left(\%\right)=\left[\left(As-An\right)/\left(Ab-An\right)\right]\times 100\%$$

As was recorded as the absorbance of REO. The absorbance of blank solution using distilled water to replace REO was recorded as *Ab*. *An* was recorded as the absorbance of control solution in the absence of H_2_O_2_. Experiments were repeated in triplicate.

#### 2.5.4. Superoxide Anion Radical Scavenging Activity of Rosemary Essential Oil

Superoxide anion radical scavenging ability of REO was assessed using the method previously reported by Siddhuraju [16]. A 0.1 M Tris-HCl buffer solution (4.5 mL, pH 8.2) was mixed with REO (1 mL) at 62.5–1000 μg/mL. These mixtures were combined with 10 mM pyrogallol solution (0.1 mL) and left at room temperature for 30 min, then immediately combined with sample, and finally, the absorbance was detected at 320 nm. Superoxide anion clearance was calculated with the following formula:

$$Superoxide\;anion\;radical\;scavenging\;rate\;\left(\%\right)=\left[\left(A_{0}-A_{S}\right)/A_{0}\right]\times 100\%$$

As was recorded as the absorbance of REO. *A*_0_ was recorded as the absorbance of blank control for pyrogallol solution. Experiments were performed in triplicate.

### 2.6. Cell Culture

A549 cells (purchased from the Cell Bank of Type Culture Collection of the Chinese Academy of Sciences, Shanghai, China) were incubated in a humidified atmosphere containing 5% CO_2_ at 37 °C, with DMEM containing 10% fetal bovine serum, 1% L-glutamine, and 1% penicillin and streptomycin. The 3–7 generations of A549 cells were used in our experiments.

### 2.7. MTT Assay

A549 cells were seeded in 96-well plates at 2 × 10^4^ cells/well overnight at 37 °C. Then, cells were incubated with REO (12.5, 25, 50, 100 μg/mL) for 24 h, with stimulation of H_2_O_2_ (100 μM) for another 4 h. Then, 5 mg/mL MTT (20 μL) was added and incubated for another 4 h at 37 °C. After that, the supernatant was substituted with 150 μL DMSO, and then, the absorbance was read at 570 nm. The Nrf2 activator Oltipraz was used as the positive standard (POS). Relative cell viability was expressed as a percentage compared to control. Experiments were performed in triplicate.

### 2.8. ROS Scavenging Activity of Rosemary Essential Oil In Vitro

A549 cells were seeded in a 96-well plate at a density of 2 × 10^4^ cells per well and incubated for 24 h. The cells were then treated with H_2_O_2_ (100 μM) and REO (12.5, 25, 50 μg/mL) in accordance with the previously described procedure. Subsequently, the supernatant was removed, and cells were incubated with 10 μM DCFH-DA (100 μL) in the dark for 30 min. Then, cells were washed twice with PBS, and the fluorescence intensity was recorded at an emission wavelength of 525 nm and an excitation wavelength of 488 nm. Meanwhile, cells were observed under a fluorescence microscope (Leica DMIL LED, Burladingen, Germany).

### 2.9. Assessment of Enzymatic and Non-Enzymatic Antioxidant Parameters In Vitro

A549 cells were seeded into a six-well plate at 1 × 10^6^ cells/well overnight. Subsequently, the cells were incubated with REO for 24 h, with the stimulation of H_2_O_2_ (100 μM) for another 4 h. Then, cells were collected and homogenated to measure the content of MDA, SOD, GSH, and glutathione disulfide (GSSG) by commercially available kits according to the manufacturer’s instructions (Beyotime Biotechnology, Shanghai, China).

### 2.10. Western Blot Analysis

A549 cells were seeded into a six-well plate and operated similarly as described above. Protein concentration was determined by BCA assay after cells were lysed with RIPA buffer at 4 °C for 30 min. An equal quantity of protein was loaded onto SDS-PAGE gels and transferred onto a PVDF membrane. The membranes were incubated with different primary antibodies overnight after blocking with 5% fat-free milk for 30 min, followed by incubation with HRP-conjugated secondary antibody for another hour. The protein bands were visualised by electrochemiluminescence (ECL) kit with Bio-Rad ChemiDoc™ imaging system (Bio-Rad, Hercules, CA, USA).

### 2.11. Molecular Docking

The 3D structure of 1,8-cineole (CAS: 406-82-6) was obtained from ChemSpider database for molecular simulation. The 3D structure of the Kelch domain of KEAP1 (ID: 4IQK) was downloaded from the Protein Data Bank (PDB) [17]. The process of molecular docking was simulated using AutoDock Vina [18]. The ligand and receptor files were converted to PDBQT format, with the insertion of polar hydrogen atoms and the removal of water molecules. Finally, all figures were visualized using PYMOL and Schrödinger molecular modeling software, and the docking scores were recorded as Gibbs free binding energy (ΔG) [19].

### 2.12. Statistical Analysis

Quantitative data were expressed as Mean ± SEM. Statistical comparison was performed by one-way analysis of variance followed by Tukey’s test using SPSS 20.0. Statistical tests were considered statistically significant with *p* < 0.05.

## 3. Results and Discussion

### 3.1. Results of Enzyme-Assisted Extraction Optimization by Single Factor and RSM

#### 3.1.1. Single-Factor Results

The effects of four independent variables on the yield of REO are illustrated in Supplementary Figure S1. At approximately 2.0% enzyme addition and an enzymatic pH of 5.0, the yield of REO reached a critical value. As the temperature exceeded 45 °C, the cellulase activity decreased with the increase in temperature until inactivation. Upon prolongation of the enzyme digestion time beyond 2 h, a slight decrease in the yield of REO was observed. Consequently, the optimal enzymatic digestion temperature and time for enzyme-assisted extraction of REO was determined as 45 °C and 2.0 h, respectively.

#### 3.1.2. The RSM Results, Variance Analysis, and Verification of Enzyme-Assisted Extraction

Due to four variables including enzyme amount, enzyme digestion pH, enzyme digestion temperature, and time, 29 trial experiments were performed using the Box–Behnken design model [20]. The actual yields are demonstrated in Table 2. 

The significance of each variable and the coefficient (R^2^) are also presented in Table 3. The model equation for the yield of REO can be found as follows: Y = 3.97 + 0.13 A + 0.06 B + 0.21 C + 0.15 D − 0.10 AB − 0.01 AC + 0.07 AD + 9.75E-03 BC + 0.15 BD + 0.08 CD − 0.34 A^2^ − 0.29 B^2^ − 0.36 C^2^ − 0.29 D^2^. The analysis by ANOVA for the BBD model demonstrated that the generated model was significant (*p* < 0.0001), and the residual lack of fit was not significant (*p* = 0.0596 > 0.05). From BBD results, a high R^2^ value of 0.9844 was found for the response. The adjusted and predicted R^2^ values were 0.9688 and 0.9148, respectively. Therefore, the regression model can fit well with the experimental data and reflect the effects of enzyme amount (A), enzyme digestion pH (B), enzyme digestion temperature (C), and time (D) on the yield of REO. According to the F-values in Table 3, the order of factors affecting the yield of REO is as follows: C > D > A > B. The F-value of BD (27.88) is considerably higher than those of AB (11.36), AD (6.11), and CD (7.27), indicating that the interaction effect of enzyme digestion pH and time is the most significant factor influencing the extraction rate. 

**Figure 1:**
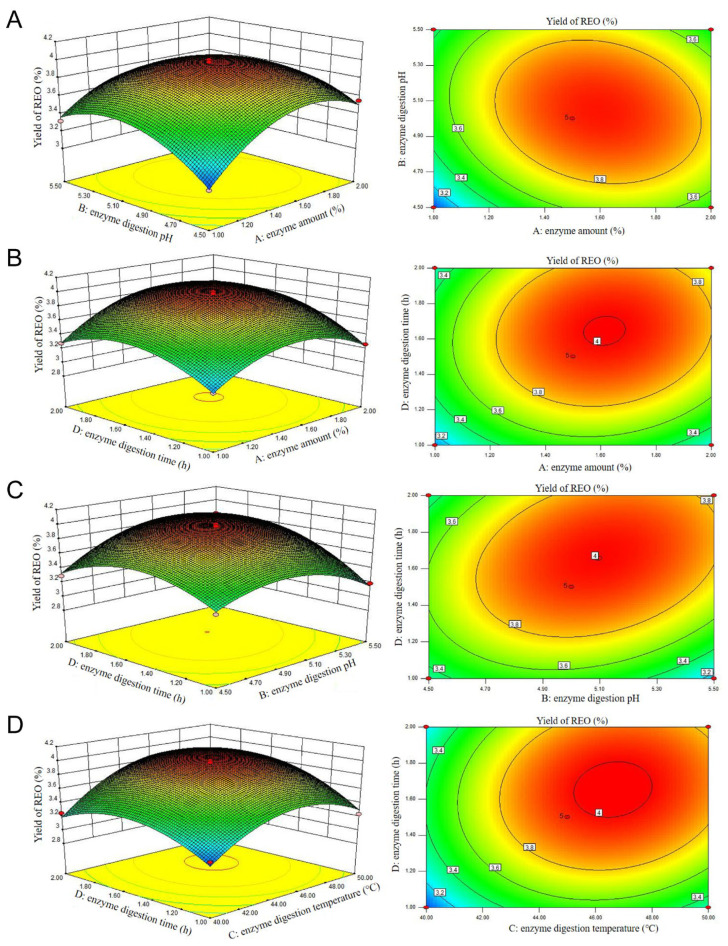
Three-dimensional and contour plots showing interaction effect of different parameters on yield: enzyme amount and enzyme digestion pH (**A**); enzyme amount and enzyme digestion time (**B**); enzyme digestion pH and time (**C**); enzyme digestion temperature and time (**D**) on yield of REO.

To further investigate the interaction effects of four independent variables on REO yield, a 3D response surface plot and contour plots obtained from the regression equation were plotted, as shown in Figure 1. The steep shape of the response surface and elliptical contours with greater curvature reflect the influence of the factor variables on the response value and the significance of the interaction between factors. A weak mutual interaction was identified in Figure 1A,B,D, which predict the relationship between the enzyme amount and enzyme digestion pH, enzyme amount and enzyme digestion temperature, and enzyme digestion temperature and time, respectively. Figure 1C illustrates a more pronounced contour line along the axis of enzyme digestion pH and enzyme digestion time, suggesting a more pronounced interaction between enzyme digestion pH and enzyme digestion time on REO yield. This finding is consistent with the results presented in Table 3. Furthermore, the adequacy of the model is demonstrated in Supplementary Figure S2.

The optimal conditions for REO (predicted yield of 4.05%) were validated through a pilot-scale test, which consisted of enzyme amount of 1.60%, enzyme digestion pH 5.09, enzyme digestion temperature 46.65 °C, and enzyme digestion time 1.69 h. These conditions were determined through an RSM analysis using the Design Expert software. The actual optimized yield of 4.10% REO was obtained under the following conditions: enzyme amount 1.60%, enzyme digestion pH 5.0, enzyme digestion temperature 46.50 °C, and enzyme digestion time 1.7 h. The obtained results were comparable to those predicted by the model, indicating the precision of the generated model. Additionally, the yield of REO obtained from hydrodistillation was considerably lower than those found by enzyme-assisted extraction (2.48 ± 0.11%). This yield is marginally higher than the average yield of 2.10% reported for most rosemary essential oils from various geographical regions [21].

### 3.2. Components of Rosemary Essential Oil

REO is a transparent yellowish liquid with unique aroma characteristics, with a specific gravity of 0.914 g/mL. A total of 37 components of REO were identified through GC–MS analysis (Figure S3), with the chemical composition presented in Table 4. The most abundant compounds of REO were 1.8-cineole (53.48%), β-pinene (20.23%), and borneol (5.21%). The constituents differed from those described by Pellegrini et al. [22], who determined the principal compound of REO to be camphor (22%) followed by α-pinene (17%), eucalyptol (16%), and borneol (12%). The components differed slightly from those reported by Jordán et al. [23], who identified camphor as the principal compound (24–36%) followed by eucalyptol (19–23%) and myrcene (9–15%). The most prevalent compounds in REO were D-camphor (63.97%), 1,8-cineole (11.52%), and α-pinene (10.08%) [24]. To the best of our knowledge, our study demonstrated that REO from Yuzhou County exhibits a high content of 1.8-cineole (53.48%), which differs from that found in other geographical regions. In accordance with the principal compound of the REO in most cases, which is present in varying proportions depending on the vegetative stage and bioclimatic conditions in different regions [21]. Agreeably, the composition and amount of REO in different plant species are subject to variation based on growing conditions, fertilization practices, harvesting season, and phenological factors [25].

### 3.3. Rosemary Essential Oil Scavenged Free Radicals in DPPH, ABTS, OH^−^, and O_2_^−^ Assay

Given that REO has been extensively employed in oxidative-related foods and biological systems, it is of significant consequence to assess the antioxidant potential of REO [21]. This study demonstrated that REO exhibited antioxidant activity in DPPH radical scavenging, with an IC_50_ of 33.61 µg/mL, in comparison to the positive control IC_50_ of VC, BHA, and BHT at 17.93, 37.24, and 48.35, respectively (Table 5). A comparable trend was observed in the ABTS free radical scavenging assay, where REO demonstrated a comparable antioxidative activity with an IC_50_ of 25.04 µg/mL, followed by BHA and BHT. With regard to the O^2−^ and OH^−^ scavenging data, it was notable that the antioxidant capacity of REO, with an IC_50_ of 50.86 µg/mL and 43.13 µg/mL, was comparable to that of BHA but higher than that of synthetic antioxidants BHT. These findings align with those of Wang et al. [26], where REO, 1,8-cineole, β-pinene, and α-pinene demonstrated DPPH radical scavenging activity with an IC_50_ value of 2.04%, 4.05%, 2.56%, and2.28% (*v*/*v*), in comparison to the positive control BHT at an IC_50_ of 2.25% (*v*/*v*). Similarly, the REO was assessed using the DPPH assay with an IC_50_ value of 77.6 μg/mL, but VC was used as a positive control with an IC_50_ value of 25.3 μg/mL [27]. REO previously demonstrated IC_50_ values of 15.10 mg/mL, 2.21 mg/mL, and 22.84 mg/mL against DPPH, ABTS, and FRAP radicals [3].

This study evaluated the superoxide and hydroxyl ion radical scavenging ability of REO for the first time. Furthermore, the antioxidant mechanism of REO has not been previously reported; thus, its antioxidative mechanism was uncovered in A549 cells in further study.

### 3.4. Effect of Rosemary Essential Oil on A549 Cell Viability

The impact of REO on cellular viability was investigated in A549 cells, which is a human lung adenocarcinoma cell model that is widely used in the field of oxidative stress and antioxidant research, typically employed as a model for pulmonary oxidative stress study. Initially, A549 cells were incubated with REO (12.5, 25, 50, 100 μg/mL) to determine the non-cytotoxic concentration. It is noteworthy that REO at 100 μg/mL resulted in a notable reduction in cell viability, which is consistent with its antitumor activity as reported in another study [28], in which REO exhibited cytotoxic effects on A549 lung cancer cells through the induction of apoptosis at high concentrations. Studies have shown that REO treatment leads to characteristic apoptotic features such as chromatin condensation, DNA fragmentation, and cell membrane blebbing. Mechanistically, REO activates the intrinsic apoptosis pathway, evidenced by the activation of caspase-3 and caspase-9. This suggests that the cytotoxicity of REO against A549 cells is primarily mediated via the mitochondrial apoptosis pathway, leading to cell death [29,30]. Consequently, the non-toxic concentration range of 12.5–50 μg/mL was selected for subsequent study (Figure S4).

The protective activity of REO against pulmonary oxidative stress was examined in H_2_O_2_-induced A549 cells. Cell viability was found to be restored remarkably with 11–35% following REO pretreatment (12.5–50 μg/mL) compared to H_2_O_2_-treated cells, indicating that REO exhibited a dose-dependent cytoprotective activity, which was consistent with extensive studies in which essential oils were shown to exert antioxidant effects on H_2_O_2_-induced A549 cells at less than 50 μg/mL (12.5, 25, 50 μg/mL) under non-cytotoxic concentrations [31,32,33]. The present study is in agreement with the results of Razavi-Azarkhiavi et al., who demonstrated that extracts of rosemary treatment strongly inhibited H_2_O_2_ damage in human lymphocytes [34].

### 3.5. Effect of Rosemary Essential Oil on the Inhibition of ROS Production in H_2_O_2_-Induced A549 Cells

**Figure 2:**
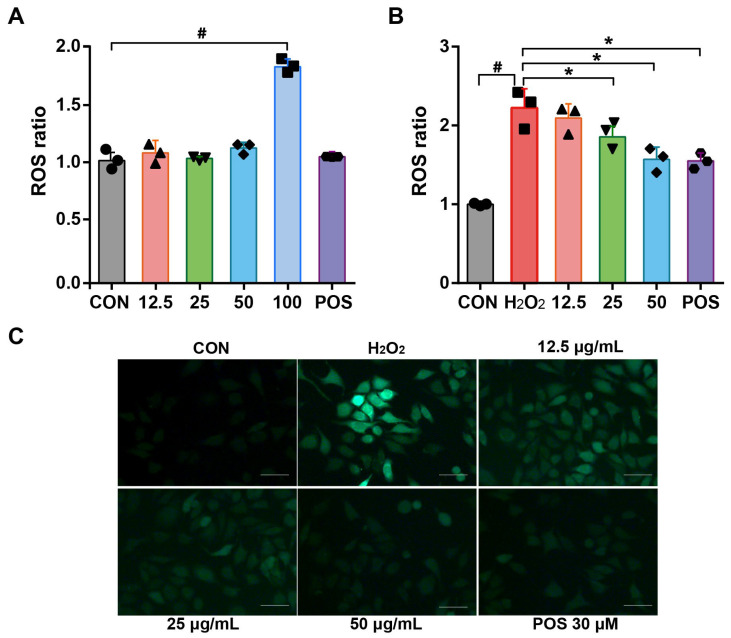
Effects of REO on intracellular ROS production in A549 cells. Cells were treated with REO samples (12.5, 25, 50, and 100 μg/mL) for 24 h and then in the absence of (**A**) or exposure (**B**) to H_2_O_2_ (100 μM) for 4 h. Intracellular ROS production was determined using the dichlorofluorescein assay with excitation at 480 nm and emission at 510 nm. (**C**) Fluorescence image of A549 cells exposed to H_2_O_2_ (100 μM) for 4 h. Oltipraz (30 μM) was used as the positive group (POS). All data shown represent the mean ± SEM of at least three independent experiments expressed as percentages (fluorescence for the control group set as 100%). ^#^ *p* < 0.05 as compared with the control group. * *p* < 0.05 as compared with the H_2_O_2_ group.

ROS, comprising H_2_O_2_, HO^−^, and O_2_, has been identified as a specific target for macromolecules (nucleic acids, lipids, proteins) and shown to disrupt the oxidative and antioxidative system, resulting in impaired cellular structure and functions [35,36]. To substantiate the antioxidative properties of REO, their ability to scavenge cellular ROS was assessed. The intracellular levels of ROS were quantified using a fluorescent probe, 2′,7′-dichlorofluorescein diacetate (DCFH-DA), which reacts with ROS to generate the fluorescent compound 2′,7′-dichlorofluorescein (DCF). As shown in Figure 2A, cells treated with REO at concentrations of 12.5, 25, and 50 μg/mL exhibited no discernible alterations in ROS levels when compared to the control group. In contrast, REO at 100 μg/mL resulted in a significant induction of ROS in response to the reduction in cell viability. However, upon exposure to H_2_O_2_, cells exhibited a pronounced induction of ROS beyond two-fold, which was in agreement with previous reports [37]. Nevertheless, the pretreatment of REO effectively neutralized the overproduction of ROS induced by H_2_O_2_. Specifically, the level of ROS decreased by 48.3% in H_2_O_2_-induced A549 cells following treatment with REO at 50 μg/mL (Figure 2B). These findings indicated that REO could alleviate oxidative stress in H_2_O_2_-induced A549 cells, and the fluorescence images of intracellular ROS effectively validated this observation (Figure 2C). This can be attributed to the phytochemical composition of REO, including 1.8-cineole (53.48%) as the main ingredient, which is known to scavenge ROS [26]. The aforementioned results indicated the potential use of REO in the treatment of oxidative-related pulmonary disorders.

### 3.6. Effect of Rosemary Essential Oil on Enzymatic and Non-Enzymatic Antioxidant Parameters in H_2_O_2_-Induced A549 Cells

**Figure 3:**
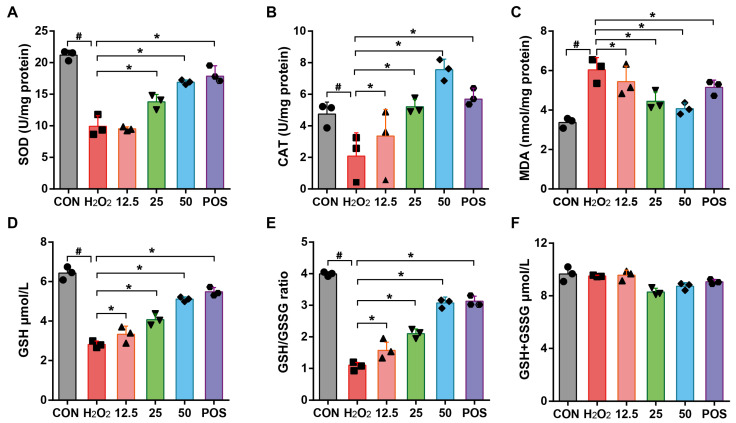
Effects of REO on oxidative stress biomarkers in A549 cells. Cells were pretreated with REO samples (12.5, 25, 50 μg/mL) for 24 h, followed by incubation with H_2_O_2_ (0.1 mM) for another 4 h. The levels of (**A**) SOD, (**B**) CAT, (**C**) MDA, (**D**) GSH, (**E**) GSH/GSSG ratio, and (**F**) GSH+GSSG were determined. Oltipraz (30 μM) was used as the positive group (POS). All data shown represent the mean ± SE of at least three independent experiments. ^#^ *p* < 0.05 as compared with the control group. * *p* < 0.05 as compared with the H_2_O_2_ group.

Oxidative stress occurs when oxidants overwhelm the antioxidant system, potentially leading to DNA damage and lipid peroxidation. The intracellular enzymatic antioxidant system, which is composed of a variety of enzymes including CAT and SOD, together with non-enzymatic antioxidant markers like MDA, GSH, and GSH/GSSG, executes numerous catalytic reactions, neutralizes free radicals, and prevents the excessive production of ROS [38,39]. To assess the protective effects of REO, H_2_O_2_ exposure resulted in a notable decline in CAT and SOD levels remarkably, while REO treatment reversed this trend dose-dependently, even back to normal levels when compared to the control (Figure 3A,B). MDA has been considered a presumptive biomarker for lipid peroxidation and has been a well-established monitor of lipid peroxidation related to oxidative stress [30]. In the present study, the level of MDA increased significantly by nearly two folds following exposure to H_2_O_2_. Conversely, the pretreatment with REO showed a statistically significant reduction in H_2_O_2_ stimulation in a dose-dependent manner. As for GSH, an abundant cellular thiol, it plays a crucial role as an antioxidant, enzyme cofactor, and modulator in oxidative stress status, accelerating the reduction of hydrogen peroxide and hydroperoxides [40]. As depicted in Figure 3C–E, cells exposed to H_2_O_2_ exhibited a significant decline in survival, accompanied by a reduction in GSH and overproduction of GSSG. Corresponding to this alternation, the level of GSH/GSSG was significantly elevated, whereas no discernible changes were observed in the GSH + GSSG level in A549 cells (Figure 3F). These results are consistent with the hypothesis that REO enhances cellular enzymes SOD and CAT, as well as GSH, which are crucial in the conversion of H_2_O_2_ into H_2_O [41]. The observation of enzymatic and non-enzymatic antioxidant alternations in this study was in accordance with the findings that demonstrated that REO prevented CCl_4_-induced oxidative stress by regulating MDA, GSH, CAT, and GSH levels in previous literature [27].

### 3.7. Effects of Rosemary Essential Oil on Nrf2 Signaling Pathway in A549 Cells

The transcription factor Nrf2 plays a pivotal role in cellular defense against toxic and oxidative insults. It is responsible for the regulation of detoxifying and antioxidant enzymes, including SOD, CAT, and GPX, which protect cells from oxidative stress [42]. Under normal circumstances, Nrf2 remains quiescent in the cytoplasm, bound to Keap1. Upon exposure to oxidative stress, Nrf2 dissociates from Keap1 and relocates to the nucleus, thereby triggering the induction of antioxidant and detoxification enzymes, including SOD, CAT, NQO1, and HO-1 [43].

**Figure 4:**
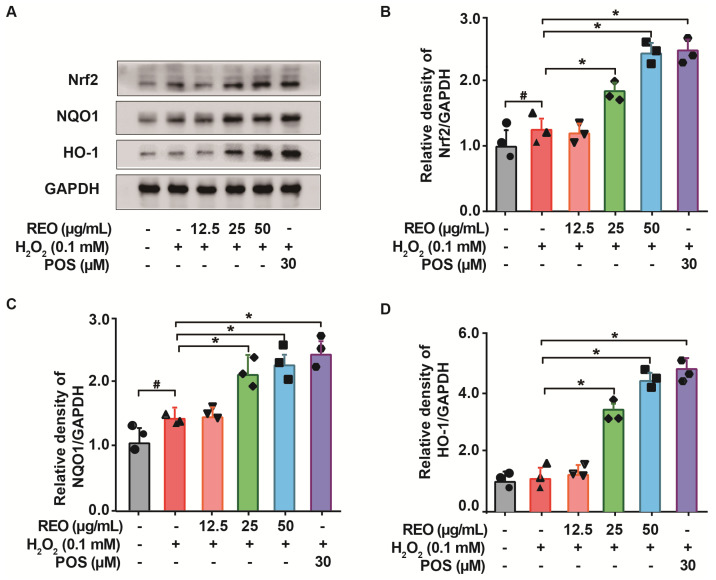
The effects of REO on the Nrf2 signaling pathway. (**A**) A549 cells were pretreated with REO samples (12.5, 25, 50 μg/mL) for 24 h, followed by incubation with H_2_O_2_ (0.1 mM) for 4 h. The total protein was lysed and extracted with RIPA buffer, and the protein levels of (**B**) Nrf2, (**C**) NQO1, and (**D**) HO-1 in each group were determined by Western blot. Blots are representative of three independent experiments. GAPDH was used as a loading control. Oltipraz (30 μM) was used as the positive group (POS). All experiments were repeated three times independently. Data are shown as mean ± SEM. ^#^ *p* < 0.05 as compared with the control group. * *p* < 0.05 as compared with the H_2_O_2_ group.

Massive evidence indicates that antioxidative stress is linked to plant-derived bioactive phytochemicals, which act as a vital antioxidant mediator for the normalization of ROS levels, the inhibition of DNA damage, and the prevention of telomere shortening via the Nrf2 signaling pathway [44]. To elucidate the mechanism accountable for the antioxidant action of REO, Western blot analysis was conducted to determine the protein levels of Nrf2 and its target genes NQO-1 and HO-1. As illustrated in Figure 4, there was a slight increase in the protein expression levels of Nrf2 and NQO1 following H_2_O_2_ treatment. This increase may be attributed to an adaptive response to the oxidative induction in accordance with previous reports [45]. Nevertheless, in comparison to the H_2_O_2_-treated group, there was an appreciable enhancement in the protein expression levels of Nrf2, NQO1, and HO-1 dose-dependently with REO, which indicates the amplified activation of the Nrf2 signaling pathway by REO treatment. Consequently, the antioxidative effect of REO may be partially attributed to the activation of the Nrf2 signaling pathway, which in turn leads to an enhancement of detoxification and antioxidants.

### 3.8. Molecular Docking

The Nrf2-Keap1 pathway is a pivotal signaling pathway involved in combating oxidative stress and boosting the cellular antioxidant capacity [35]. Inhibitors of Keap1 have the potential to disrupt the covalent interaction between Nrf2 and Keap1. This disruption releases the transcriptional machinery of Nrf2, which coordinates cellular antioxidant and detoxification processes and safeguards cells against oxidative stress-induced disorders [46]. In light of the antioxidant activity and the results of the chemical composition analysis described above, it is also important to note that the volatile aroma components in REO, especially 1.8-cineole (54.05%), are known to exhibit antioxidant biological activity [26]. With regard to the underlying mechanism of REO as an Nrf2 activator, the potential attachment between 1.8-cineole monomers and the Kelch domain of KEAP1 (PDB ID: 4IQK) was analyzed using the proposed molecular docking model.

**Figure 5:**
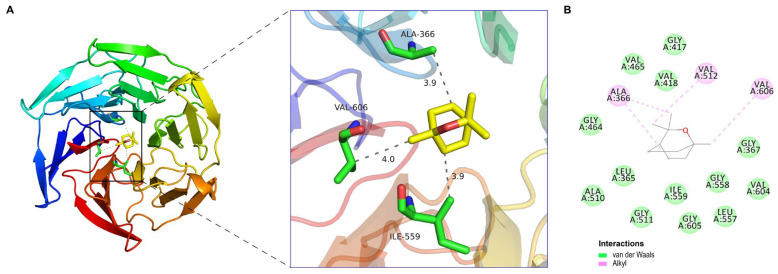
The molecular interactions of 1.8-cineole in the Kelch domain of KEAP1 (PDB ID: 4IQK). (**A**) The three-dimensional (3D) interaction diagrams; (**B**) the 2D interactions (green: van der Waals; red: alkyl) between the functional groups of the compounds with the specific amino acids.

Molecular docking based on virtual screening of 1,8-cineole-binding protein and the Kelch domain of KEAP1 determined the binding affinity values of the bioactive ligands against the active sites of the protein target by AutoDock Vina software. The lowest Gibbs free binding energy estimated as ∆G = −5.5 kcal/mol (<0 kcal/mol), further confirmed the stability of 1.8-cineole docked into the Kelch domain of KEAP1. As shown in Figure 5A, the critical amino acids engaged in the molecular interactions between 1.8-cineole and the Kelch domain of KEAP1, formed alkyl interactions with ALA366, VAL606, and ILE559, as identified by PYMOL software. As shown in Figure 5B, the Schrödinger molecular modeling software revealed that 1.8-cineole formed alkyl interactions with the KEAP1 protein residues ALA366, VAL512, and VAL606. Additionally, it formed Van der Waal’s interactions with GLY417, VAL465, VAL418, GLY464, LEU365, ALA510, GLY511, ILE559, GLY605, GLY558, LEU557, VAL604, and GLY367. The results indicated that 1.8-cineole interacted with the Kelch domain of KEAP1 stably through the formation of alkyl interactions with ALA366 and VAL606. Molecular docking studies have demonstrated that 1.8-cineole exhibits a promising competitive interaction with the Keap1 protein. The outcomes indicated that 1.8-cineole may contribute to REO’s antioxidant ability via the activation of the Nrf2 signaling pathway.

Indeed, it is very difficult to attribute the antioxidant effect of a total essential oil to a single or a few active principles. The ongoing study will further explore the results of major and minor compounds contributing to the antioxidant activity of the REO.

## 4. Conclusions

In this study, the enzyme-assisted extraction of REO was optimized using response surface methodology with the conditions of enzyme amount (1.60%), enzyme digestion pH (5.0), enzyme digestion temperature (46.50 °C), and enzyme digestion time (1.7 h). In addition, REO was shown to suppress lipid peroxidation and restore the activity of antioxidant and metabolic enzymes via activation of the Nrf2 pathway in H_2_O_2_-induced A549 cells. Furthermore, molecular docking verified that 1.8-cineole may be a promising competitive interaction with Keap1 as an Nrf2 inducer. To sum up, REO can be considered a valuable natural antioxidant agent in the food and pharmaceutical industries.

## Figures and Tables

**Table 1:** The variables and levels used in Box–Behnken design experiment.

Variable	Low Level (−1)	Central Level (0)	High Level (+1)
A (enzyme amount/%)	1.5	2.0	2.5
B (enzyme digestion pH)	4.5	5.0	5.5
C (enzyme digestion temperature/°C)	40	45	50
D (enzyme digestion time/h)	1.5	2.0	2.5

**Table 2:** Results from Box–Behnken design model with four variables.

RUN	A	B	C	D	Actual Yield (%)
1	−1	0	0	1	3.28
2	−1	−1	0	0	3.03
3	0	0	1	1	3.71
4	−1	1	0	0	3.32
5	0	0	1	−1	3.24
6	0	0	0	0	3.97
7	0	0	0	0	3.98
8	1	−1	0	0	3.55
9	0	0	−1	−1	3.09
10	0	0	−1	1	3.25
11	1	0	1	0	3.56
12	0	−1	0	−1	3.29
13	1	0	0	−1	3.26
14	−1	0	1	0	3.39
15	1	0	−1	0	3.17
16	0	0	0	0	3.93
17	1	0	0	1	3.71
18	0	0	0	0	3.98
19	−1	0	0	−1	3.12
20	0	1	0	1	3.80
21	0	0	0	0	4.00
22	0	−1	−1	0	3.03
23	−1	0	−1	0	2.95
24	0	1	0	−1	3.19
25	1	1	0	0	3.45
26	0	1	1	0	3.64
27	0	1	−1	0	3.09
28	0	−1	1	0	3.54
29	0	−1	0	1	3.29

**Table 3:** Analysis of variance for the Box–Behnken design model results.

Source	Sum of Squares	Degree of Freedom	Mean Square	*F* Value	*p* Value	Inference
Model	2.96	14	0.21	63.16	<0.0001	**
A	0.22	1	0.22	64.69	<0.0001	**
B	0.047	1	0.047	14.15	0.0021	**
C	0.51	1	0.51	153.49	<0.0001	**
D	0.28	1	0.28	83.65	<0.0001	**
AB	0.038	1	0.038	11.36	0.0046	**
AC	6.76 × 10^−4^	1	6.76 × 10^−4^	0.2	0.66	NS
AD	0.02	1	0.02	6.11	0.0269	*
BC	3.80 × 10^−4^	1	3.80 × 10^−4^	0.11	0.7411	NS
BD	0.093	1	0.093	27.88	0.0001	**
CD	0.024	1	0.024	7.27	0.0174	*
A^2^	0.76	1	0.76	227.68	<0.0001	**
B^2^	0.55	1	0.55	163.84	<0.0001	**
C^2^	0.83	1	0.83	247.52	<0.0001	**
D^2^	0.54	1	0.54	162.01	<0.0001	**
Residual	0.047	14	3.35 × 10^−3^			
Lack of fit	0.044	10	4.36 × 10^−3^	5.38	0.0596	NS
Pure error	3.25 × 10^−3^	4	8.11 × 10^−4^			
Cor total	3.01	28				
Std. Dev. = 0.058	Mean = 3.44	C.V.% = 1.68	R^2^ = 0.9844	R^2^ _Adj_ = 0.9688	R^2^ _Pred_ = 0.9148	

A: enzyme amount, B: enzyme digestion pH, C: enzyme digestion temperature, D: enzyme digestion time, Mean Square: the average of the squared differences between the observed and predicted values, Sum of Squares: the total of the squared differences between the observed and predicted values, Cor total: totals of all information corrected for the mean, C.V.: coefficient of variation. * *p* ≤ 0.05, ** *p* ≤ 0.01, NS: not significant.

**Table 4:** Components and content of rosemary essential oil.

NO ^a^.	Rt (Min)	Name	CAS Number	RI ^b^ (NIST)	RI ^c^ (C5~C40)	Content ^d^ (%)
1	7.38	Thujene	2867-05-2	911	912	0.28 ± 0.01
2	8.40	α-Pinene	7785-70-8	917	916	0.97 ± 0.28
3	8.72	Tricyclene	508-32-7	919	922	0.03 ± 0.01
4	9.72	Camphene	79-92-5	933	936	0.18 ± 0.05
5	10.26	1,5-Heptadiene, 2,5-dimethyl-3-methylene-	124-19-6	949	953	0.28 ± 0.08
6	10.96	Aniline	124-13-0	954	958	1.04 ± 0.12
7	13.45	Sabinene	3387-41-5	961	964	0.12 ± 0.09
8	13.74	β-Pinene	127-91-3	964	965	20.23 ± 2.36
9	14.02	Myrcene	123-35-3	981	980	0.13 ± 0.06
10	14.19	3-Octanol	589-98-0	994	997	0.17 ± 0.08
11	14.35	p-Cymene	99-87-6	1021	1025	0.15 ± 0.01
12	14.57	1,8-Cineole	470-82-6	1028	1030	53.48 ± 4.37
13	14.69	D-Limonene	5989-27-5	1030	1034	3.25 ± 1.35
14	14.75	1-Hexanol, 2-ethyl-	104-76-7	1031	1035	0.25 ± 0.13
15	15.17	1,3,6-Octatriene, 3,7-dimethyl-, (Z)-	3338-55-4	1039	1042	0.07 ± 0.01
16	15.49	Linalool	78-70-6	1104	1108	0.08 ± 0.01
17	15.56	Bicyclo[2.2.1]heptane, 1,7,7-trimethyl-	464-15-3	1140	1144	4.25 ± 0.89
18	15.63	D-Camphor	464-49-3	1144	1149	1.53 ± 0.45
19	15.78	Pinocarvone	24-41-3	1164	1161	0.36 ± 0.09
20	16.18	Borneol	464-43-7	1166	1166	5.21 ± 0.26
21	16.32	Benzofuran, 4,5,6,7-tetrahydro-3,6-dimethyl-	494-90-6	1169	1170	0.20 ± 0.01
22	16.44	dl-Menthol	89-78-1	1173	1174	3.15 ± 0.97
23	16.69	α-Terpineol	98-55-5	1191	1196	0.10 ± 0.01
24	16.82	D-Verbenone	80-57-9	1200	1202	0.14 ± 0.10
25	16.87	Terpinen-4-ol	562-74-3	1206	1205	0.17 ± 0.01
26	17.17	4-Acetylbenzoic acid	586-89-0	1236	1236	0.34 ± 0.21
27	17.42	Pulegone	89-82-7	1244	1246	1.23 ± 0.12
28	17.55	Piperitone	89-81-6	1252	1258	0.68 ± 0.05
29	19.88	(+)-Cyclosativene	22469-52-9	1364	1369	0.05 ± 0.01
30	19.94	α-ylangene	14912-44-8	1406	1408	0.21 ± 0.08
31	20.35	β- Caryophyllene	87-44-5	1417	1420	0.07 ± 0.01
32	21.20	Elemene	11029-06-4	1445	1449	0.32 ± 0.13
33	22.10	α-Caryophyllene	6753-98-6	1452	1455	0.18 ± 0.05
34	22.39	γ-Muurolene	30021-74-0	1474	1478	0.05 ± 0.01
35	22.58	Espatulenol	6750-60-3	1571	1576	0.18 ± 0.11
36	22.69	Globulol	51371-47-2	1575	1577	0.15 ± 0.08
37	22.99	Caryophyllene oxide	1139-30-6	1578	1581	0.09 ± 0.01

^a^ Components are noted in order of their inclusion in the HP 5 polar column. ^b^ Retention indices obtained from NIST 17. ^c^ Retention indices on an HP-5 MS column obtained experimentally using a homologous n-alkanes series (C7–C40). ^d^ Results from triplicates are presented as mean ± standard deviation.

**Table 5:** Radical scavenging activity of rosemary essential oil (IC_50_ µg/mL).

Sample	DPPH	ABTS	O_2_^−^	OH^−^
REO	33.61 ± 0.24	25.04 ± 1.33	50.86 ± 3.66	43.13 ± 6.32
VC	17.93 ± 0.30	15.52 ± 1.45	30.27 ± 3.89	23.95 ± 3.45
BHA	37.24 ± 0.45	29.31 ± 2.56	58.50 ± 4.27	45.57 ± 5.71
BHT	48.35 ± 0.67	35.75 ± 2.78	67.63 ± 5.89	47.25 ± 5.18

REO: Rosemary essential oil; VC: Vitamin C; BHA: Butylated hydroxyanisole; BHT: Butylated hydroxytoluene.

## Data Availability

The original contributions presented in the study are included in the article, further inquiries can be directed to the corresponding authors.

## Supplementary Materials

The following supporting information can be downloaded at: https://www.mdpi.com/article/10.3390/molecules29143382/s1, Figure S1: Effect of enzyme amount (A), enzyme digestion pH (B), enzyme digestion temperature (C) and time (D) on the yield of enzyme-assisted extraction of Rosmarinus officinalis L. essential oil (REO); Figure S2: Model adequacy checking was obtained by normal plot (A), run number (B), versus residuals, and predicted versus actual value (C); Figure S3: GC-MS analysis of REO; Figure S4: Effects of REO on A549 cell viability. (A) Cells were incubated with REO (12.5, 25, 50, and 100 μg/mL) for 24 h. Cell viability was estimated using MTT assay. Values were expressed as percentages (absorbance for control cells as 100%). (B) Cells were treated with REO (12.5, 25, 50 μg/mL) for 24 h, followed by incubation with H_2_O_2_ (100 μM) for another 4h. The protective effect against H_2_O_2_ was determined by MTT assay. Oltipraz (30 μM) was used as the positive control (POS). Data were expressed as percentages, considering the absorbance for control cells as 100%. All data shown represent the mean ± SEM of at least three independent experiments. # *p* < 0.05 as compared with the control group. * *p* < 0.05 as compared with the H_2_O_2_ group.
