# Supplementary material for: Optimization of Enzyme-Assisted Extraction of Rosemary Essential Oil Using Response Surface Methodology and Its Antioxidant Activity by Activating Nrf2 Signaling Pathway
Source: Molecules. 2024 Jul 18;29(14):3382. doi: 10.3390/molecules29143382 (PMC11279388; doi:10.3390/molecules29143382)
Supplement: Supplementary file 1 [file molecules-29-03382-s001.zip › molecules-3029129-supplementary.pdf]

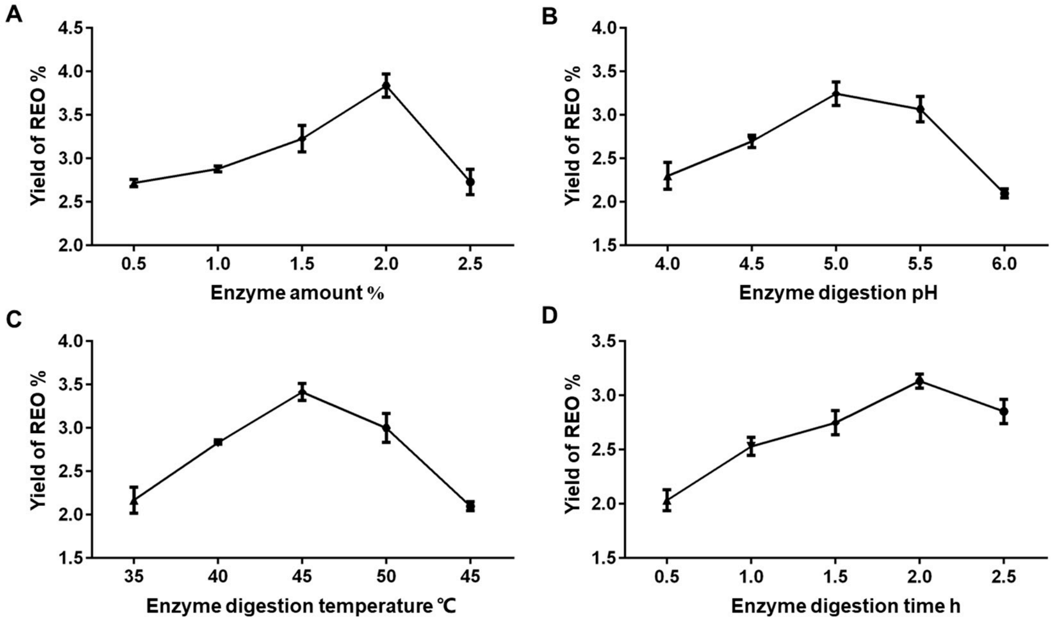

Figure S1. Effect of enzyme amount (A), enzyme digestion pH (B), enzyme digestion temperature (C) and time (D) on the yield of enzyme-assisted extraction of *Rosmarinus officinalis* L. essential oil (REO).

The typical plot (Fig. S2 A) displays a linear trend indicative of a normal distribution, independent of each variable. The residuals versus run number plot (Fig. S2 B) demonstrates a random distribution of values ranging from + 3 to -3 indicating a quadratic model correlation between the four independent variables and REO yield. The plots between predicted versus actual values are shown in Fig. S3 C. This plot presents a straight line, indicating that the generated model was constructed to predict accurately in comparison to the actual response values.

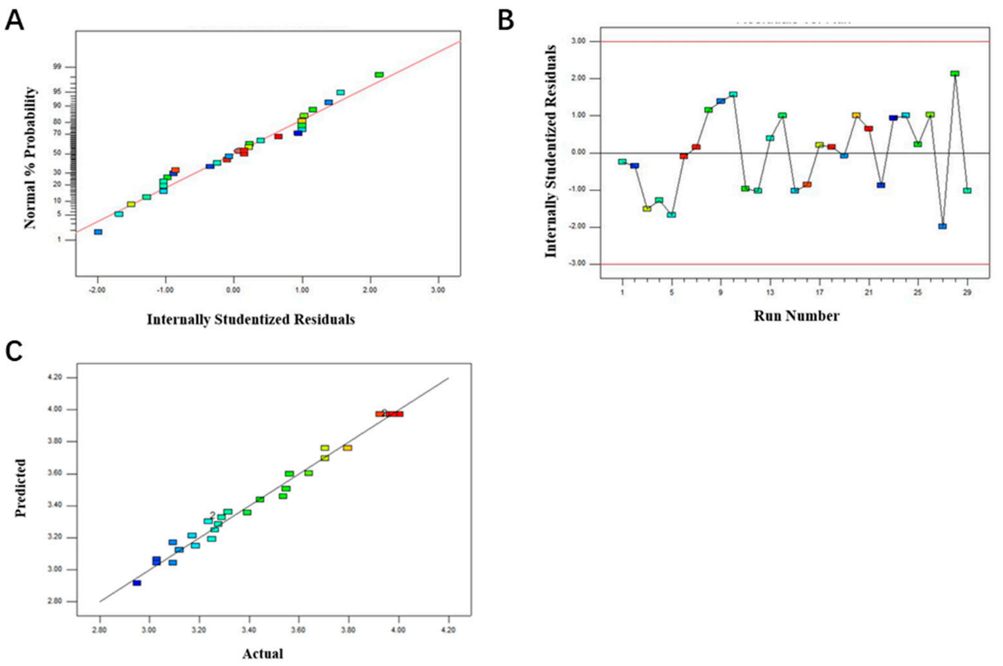

Figure S2. Model adequacy checking was obtained by normal plot (A), run number (B), versus residuals, and predicted versus actual value (C).

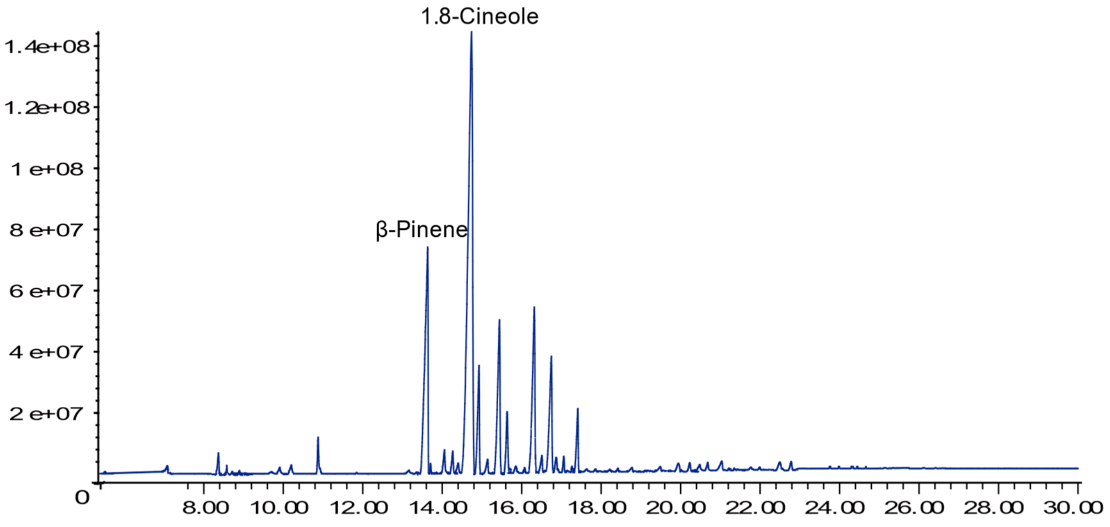

Figure S3. GC-MS analysis of REO.

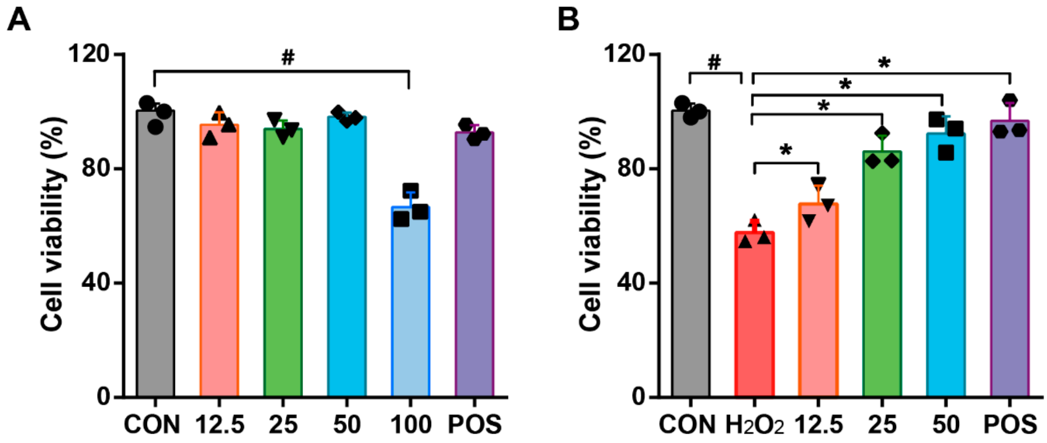

Figure S4. Effects of REO on A549 cell viability. (A) Cells were incubated with REO (12.5, 25, 50, and 100 μg/mL) for 24 h. Cell viability was estimated using MTT assay. Values were expressed as percentages (absorbance for control cells as 100%). (B) Cells were treated with REO (12.5, 25, 50 μg/mL) for 24 h, followed by incubation with H_2O_2 (100 μM) for another 4h. The protective effect against H_2O_2 was determined by MTT assay. Oltipraz (30 μM) was used as the positive control (POS). Data were expressed as percentages, considering the absorbance for control cells as 100%. All data shown represent the mean ± SEM of at least three independent experiments. $^{\#}$ $p < 0.05$ as compared with the control group. * $p < 0.05$ as compared with the H_2O_2 group.
